# Estimates of inpatient admission rates and associated costs for infants before and after China’s universal two-child policy

**DOI:** 10.1186/s12913-022-07571-9

**Published:** 2022-02-08

**Authors:** Menghan Shen, Xiaoxia Liang, Yushan Wu, Shixin Fang

**Affiliations:** 1 Center for Chinese Public Administration Research, School of Government, Sun Yat-sen University, Guangzhou, 510275 China; 2 Department of Infectious Diseases and Public Health, Jockey Club College of Veterinary Medicine and Life Sciences, City University of Hong Kong, Kowloon Tong, Hong Kong, 999077 Hong Kong; 3 Institute of Higher Education, Shanghai Center for Innovation and Governance, Fudan University, 220 Handan Road, Shanghai, 200433 China

**Keywords:** China, Economic burden, Public health insurance, Gender, Infant health, Universal two-child policy

## Abstract

**Background:**

China implemented a universal two-child policy in 2015. It is important to understand infants’ medical utilization in the context of this policy to inform health policies and resource allocation.

**Methods:**

This study utilized a 20% random sample of administrative data from China’s Urban and Rural Basic Medical Insurance (URBMI) in one of the largest southern Chinese cities from January 2015 to June 2018. Ordinary least squares models were used to estimate changes in inpatient admission rates and costs for infants between 0 and 6 months old after the implementation of China’s universal two-child policy.

**Results:**

The overall inpatient admission rate was 27.2% in 2015 and 31.3% in 2017. Compared with 2015, there was an increase in inpatient admission rates for infants 1 month old or younger (coef = 0.038, 95% CI = 0.029 to 0.047, *p* < .001) and infants 6 months old or younger (coef = 0.041, 95% CI = 0.030 to 0.052, *p* < .001) in 2017. The increase was larger for male infants than for female ones. The average inpatient admission cost was 8412.3 RMB ($1320.61) (SD = 15,088.2). There was no increase in inpatient admission costs overall. The average length of hospital stay was 7.3 days, the probability of going to a tertiary hospital was 76.2%, and the share of out-of-pocket costs was 53.0% for all diseases.

**Conclusion:**

After the implementation of the universal two-child policy in China, there was a significant increase in inpatient admission rates, especially for male infants. The overall associated costs did not change, but the increase in admission rates caused additional economic burdens for families and for social health insurance. Understanding the healthcare utilization of infants in the universal two-child period can provide insight for healthcare resource allocation in a time of dramatic changes in population policy.

**Supplementary Information:**

The online version contains supplementary material available at 10.1186/s12913-022-07571-9.

## Introduction

Over the last 70 years, China has made dramatic adjustments to its population policy. A strict one-child policy was in effect from 1979 to 2013 in urban areas to address overpopulation and its related socioeconomic challenges. Because of the shrinking labor force and rapidly aging society, China gradually relaxed the one-child policy and allowed couples to have a second child if both of the parents were an only child; from November 2013, couples were allowed to have a second child if either of the parents was an only child (the selective two-child policy). In October 2015, China implemented a universal two-child policy, signaling a relaxation of the once tightly controlled one-child policy [1]. Before the universal two-child period, if couples not eligible to have a second child violated the one-child policy and had a second child, they would be fined and may lose employment at the formal sector, and the child could not obtain Hukou status (household registration system, an important determinant of differential welfare in China), and would not have access to social benefits such as education and healthcare. Under the universal two-child policy, the second child can obtain Hukou and are eligible for social welfare and parents no longer fear losing jobs [2, 3]. The official announcement of the universal two-child policy stimulated fertility in the following 2 years. The number of live births per year increased from 16.55 million in 2015 to 17.86 million in 2016 and 17.23 million in 2017 [4]. Individual-level delivery records reveal an increase of 5.4 million births attributed to the universal two-child policy between October 2016 and December 2017 as compared with January 2014 to September 2016 [5].

Changes in women’s pregnancy age and birth outcomes have been reported in connection with changes in China’s population policy [4, 5]. More specifically, the universal two-child policy targeted women of reproductive age who had a previous delivery. This group was estimated to be about 90 million women, and among them, an estimated 60% were over 35 and 50% over 40 [6]. A study that used national data with county-level monthly aggregated data and individual level delivery information records has estimated that the two-child policy was associated with a 68.2% increase in births by women of advanced maternal age (aged 35 or older) from a baseline level of 8.5% [5]. Another study using a hospital-based birth defect surveillance system in Zhejiang Province found an increase of 85.7% of women with advanced maternal age from a baseline of 8.5%. With an increase in births to mothers of advanced age, there is a concern that there would be an increase in maternal and child health problems due to the established relationship between advanced maternal age and various risk factors of maternal health and newborn health [7, 8]. In addition, the characteristics of the parents that would be giving birth after the universal two-child policy may be different from parents that gave birth before the universal two-child policy. More specifically, those that respond to the universal two-child policy by having a second child may have higher or lower socioeconomic status as compared with people who only have one child, thus changing the socioeconomic status of the families giving birth before and after the universal two-child period.

However, literature on the association between the two-child policy and infant health outcomes is scant, and current literature has reported mixed relationships between the two-child policy and infant health outcomes. One study using national data found no concurrent preterm births [5], another using data from the Zhejiang province found an increase in the probability of birth defects [9]. In addition, because of the availability of the data, these studies have generally discussed obstetric outcomes; so far, the nonobstetric medical utilization of infants has not been described.

To partially fill this gap in the literature, in this study, we estimated changes in inpatient admission rates and associated costs for infants 0 to 6 months old after the implementation of China’s universal two-child policy. We utilized a 20% random sample of administrative data from China’s Urban and Rural Basic Medical Insurance (URBMI) to examine these changes. In addition, we conducted subgroup analyses to understand whether changes in inpatient admission rates and costs differed by infant gender, age, and disease type. Our findings have policy implications for healthcare utilization prediction and healthcare resource allocation in a time of dramatic changes in population policy.

## Methods

### Data

We utilized a 20% random sample of administrative data from China’s URBMI in one of the largest southern cities (referred to as City A for anonymity) from January 2015 to June 2018. The URBMI in China is a voluntary medical insurance scheme accessible to urban and rural residents and subsidized by the Chinese government [10]. It provides healthcare to individuals who are not covered by the country’s Urban Employee Basic Medical Insurance (UEBMI), including children. Our dataset contains pseudonymized enrollment and medical claim records from City A. The enrollment records include basic demographic information, such as gender, age, and hukou status (the Chinese household registration system). The medical claim records represent individual enrollees’ insurance claims and include the date of visit, provider facility name, provider type and level, the region of residence, diagnosis code (International Classification of Diseases [ICD-10] code), cost, cost by payment source (out-of-pocket and reimbursed), and cost by category (e.g., medicine, treatment, checkup, operation/procedure, other).

Our dataset likely captures a significant portion of inpatient visits among infants under the age of 6 months, even though participation in the URBMI scheme is not mandatory for residents of City A. Because of the low cost of enrollment and generous government subsidies, 95% of residents in China are enrolled either in URBMI or UEBMI [11]. However, because parents need to apply for URBMI in person for their children, the enrollment rate of URBMI is comparatively low for very young children [12]. For example, in our dataset, among children aged 0–1 years, using the number of enrollees as the numerator and the population count of children born in City A as the denominator, only 64% born in the city are enrolled in URBMI. However, parents can retrospectively apply for URBMI coverage before the infant turns 6 months old. In other words, if an infant is hospitalized at 2 months old, the parents can apply for URBMI after the inpatient visit and before the infant turns 6 months old, and URBMI will cover the cost. Because hospitalization of infants usually imposes an economic burden on a household without URBMI coverage, families with infants admitted as inpatients under the age of 6 months are very likely to apply for URBMI.

To correctly estimate the rate of inpatient admissions, we used the population count of children born in City A as the denominator. The population count and gender of children born in City A were obtained from the provincial health statistics yearbook [13].

### Variables

Medical care utilization included the inpatient admission rate, costs per visit, length of stay per visit, and whether the visit took place in a tertiary hospital.

To calculate the inpatient admission rate, essentially, we counted the total number of inpatient admissions that took place for the child and divided it by the total number of births in the city in that year. Inpatient admissions include hospitalizations at primary care facilities as well as secondary and tertiary hospitals. In operation, as we conducted the main analysis at the individual level, and there were cases of multiple admissions, the outcome variable was the number of admissions for an individual. For example, if the infant visited the hospital twice in the period of interest, the outcome variable was assigned two, and if infants who were born in that year (2015 or 2017) but did not have any inpatient visit for the first 6 months of their life, the outcome variable was assigned zero. For robustness analysis, we changed the outcome variable to a dummy that equals one if the individual has ever had an inpatient visit, and zero if the individual never had any inpatient visit.

Inpatient costs were measured in terms of the average cost per visit, the average out-of-pocket cost per visit, and the average reimbursed cost per visit. In addition, inpatient medical costs were broken down into subtypes (medicine, treatment, checkup, operation/procedure, and other). All costs were measured in RMB and converted to USD ($) at an exchange rate of RMB/USD = 6.37. Out-of-pocket costs include deductibles, coinsurance, copayments, and costs of drugs, treatments, or tests not covered by URBMI.

### Analysis

As the universal two-child policy was announced in October 2015, births prior to June 2016 (9 months after the announcement of the universal two-child policy) were usually considered part of the baseline period. We treated January 2015 to December 2015 as the baseline period and January 2017 to December 2017 as the post-period.

We compared inpatient admission rates and associated costs for infants aged 0–6 months between 2015 and 2017. As this is a before-and-after comparison, the assumption of the study is that no other things had changed dramatically between 2015 to 2017. To the best of our knowledge, there was no significant increase in health insurance policy between 2015 to 2017 or dramatic differences in hospital resources, thus this assumption is reasonable.

We constructed ordinary least squares models to compare means between 2015 and 2017. We took the log of costs to account for skewness. It is important to add in controls to account for changes that can affect infant health but are not related to the two child policy. In our design, we did not add controls because we did not have additional individual-level information in the dataset. We also conducted this analysis by gender and for each of the five most diagnosed diseases in our sample. We used robust standard errors to correct for heteroskedasticity. A *p*-value of less than .05 was considered statistically significant. All statistical analyses were conducted in STATA 16. Ethical approval for the study (SBRE-19-768) was obtained from the Chinese University of Hong Kong.

## Results

### Inpatient admission rates

Table 1 presents results on the number of births and inpatient admission rates by gender and infant age. Compared with 2015, there was an increase in the number of infants born in 2017 overall (90,595 vs. 155,445) and for both male infants (49,325 vs. 81,950) and female infants (41,270 vs. 73,495). There was a 71.58% increase in births overall, a 66.14% increase in male births, and a 78.08% increase in female births.

**Table 1:** Inpatient admission rates within 1 month and 6 months of birth

	Total	2015	2017	2017 versus 2015		
	Mean (SD)	Mean (SD)	Mean (SD)	Estimate	CI	*p*
Total						
Within 1 month	0.249 (0.498)	0.225 (0.477)	0.263 (0.509)	0.038***	(0.029, 0.047)	<.001
Within 6 months	0.298 (0.594)	0.272 (0.58)	0.313 (0.602)	0.041***	(0.030, 0.052)	<.001
*N*	246,040	90,595	155,445			
Male						
Within 1 month	0.262 (0.510)	0.232 (0.487)	0.281 (0.523)	0.049***	(0.036, 0.061)	<.001
Within 6 months	0.323 (0.625)	0.289 (0.602)	0.343 (0.637)	0.054***	(0.039, 0.070)	<.001
*N*	131,275	49,325	81,950			
Female						
Within 1 month	0.233 (0.483)	0.216 (0.464)	0.243 (0.493)	0.028***	(0.015, 0.041)	<.001
Within 6 months	0.270 (0.556)	0.253 (0.551)	0.280 (0.559)	0.027***	(0.012, 0.042)	<.001
*N*	114,765	41,270	73,495			

Notes: An ordinary least squares regression is used to compare the inpatient admission rates for infants born in 2015 versus 2017

^**^p < .05

^***^p < .01

Compared with 2015, there was an increase in inpatient admission rates of infants 1 month old or younger (coef = 0.038, 95% CI = 0.029 to 0.047, *p* < .001) and infants 6 months old or younger (coef = 0.041, CI = 0.030 to 0.052, *p* < .001) in 2017. The increase was larger for male infants (within 1 month old: coef = 0.049, CI = 0.036 to 0.061; within 6 months old: coef = 0.054, CI = 0.039 to 0.070, all *p* < .001) than for female ones (within 1 month old: coef = 0.028, CI = 0.015 to 0.041; within 6 months old: coef = 0.027, CI = 0.012 to 0.042, all *p* < .001).

Table 2 presents results on inpatient admission rates for infectious and noninfectious diseases. Compared with 2015, inpatient admission rates increased for both infectious diseases (coef = 0.013, CI = 0.007 to 0.018, *p* < .001) and noninfectious diseases (coef = 0.028, CI = 0.019 to 0.037, *p* < .001). In both categories, the growth was larger for male infants (infectious: coef = 0.015, CI = 0.007 to 0.024; noninfectious: coef = 0.039, CI = 0.026 to 0.052, all *p* < .001) than for female ones (infectious: coef = 0.010, CI = 0.003 to 0.018, *p* = .005; noninfectious: coef = 0.016, CI = 0.003 to 0.029, *p* = .013).

**Table 2:** Inpatient admission rates by disease type

	Total	2015	2017	2017 versus 2015		
	Mean (SD)	Mean (SD)	Mean (SD)	Estimate	CI	*p*
Infectious diseases						
Total	0.073 (0.303)	0.065 (0.285)	0.078 (0.313)	0.013***	(0.007, 0.018)	<.001
Male	0.086 (0.331)	0.076 (0.311)	0.092 (0.343)	0.015***	(0.007, 0.024)	<.001
Female	0.059 (0.267)	0.052 (0.25)	0.063 (0.277)	0.010***	(0.003, 0.018)	0.005
Noninfectious diseases						
Total	0.225 (0.503)	0.207 (0.497)	0.235 (0.506)	0.028***	(0.019, 0.037)	<.001
Male	0.236 (0.520)	0.212 (0.509)	0.251 (0.527)	0.039***	(0.026, 0.052)	<.001
Female	0.211 (0.482)	0.201 (0.482)	0.217 (0.482)	0.016**	(0.003, 0.029)	.013

Notes: An ordinary least squares regression is used to compare the inpatient admission rates for infants born in 2015 versus 2017

^**^p < .05

^***^p < .01

Table 3 presents results on inpatient admission rates for each of the five most commonly diagnosed disease types in our sample: jaundice, pneumonia, preterm birth/small size for gestational age, bronchitis, and neonatal aspiration syndrome. They account for 12.2, 4.7, 3.7, 0.8, and 0.8% of hospitalizations for infants within 6 months old. In 2017, there was a rise in inpatient admission rates for jaundice (coef = 0.022, CI = 0.016 to 0.029, *p* < .001) and pneumonia (coef = 0.012, CI = 0.008 to 0.016, *p* < .001) and a decrease for bronchitis (coef = − 0.002, CI = − 0.004 to − 0.001, *p* = .010). Admission rates for preterm birth/small size for gestational age and neonatal aspiration syndrome did not appear to change.

**Table 3:** Inpatient admission rates by disease (top 5 diseases)

	Total	2015	2017	2017 versus 2015		
	Mean (SD)	Mean (SD)	Mean (SD)	Estimate	CI	*p*
Jaundice						
Total	0.122 (0.369)	0.108 (0.349)	0.130 (0.38)	0.022***	(0.016, 0.029)	<.001
Male	0.127 (0.380)	0.110 (0.362)	0.138 (0.390)	0.027***	(0.018, 0.037)	<.001
Female	0.115 (0.356)	0.104 (0.332)	0.121 (0.368)	0.017***	(0.007, 0.027)	<.001
Pneumonia						
Total	0.047 (0.238)	0.039 (0.216)	0.051 (0.250)	0.012***	(0.008, 0.016)	<.001
Male	0.055 (0.261)	0.046 (0.236)	0.061 (0.275)	0.015***	(0.008, 0.021)	<.001
Female	0.037 (0.209)	0.031 (0.189)	0.041 (0.219)	0.010***	(0.004, 0.015)	<.001
Preterm birth/small for gestational age						
Total	0.037 (0.192)	0.038 (0.196)	0.037 (0.189)	−0.001	(−0.005, 0.002)	.546
Male	0.037 (0.192)	0.037 (0.193)	0.037 (0.191)	0.001	(− 0.004, 0.006)	.723
Female	0.037 (0.191)	0.039 (0.199)	0.036 (0.186)	−0.003	(− 0.009, 0.002)	.203
Bronchitis						
Total	0.008 (0.097)	0.010 (0.108)	0.007 (0.090)	−0.002***	(−0.004, − 0.001)	.010
Male	0.010 (0.107)	0.011 (0.118)	0.009 (0.100)	−0.002	(−0.005, 0.000)	.110
Female	0.006 (0.084)	0.008 (0.095)	0.005 (0.077)	−0.002**	(−0.005, − 0.000)	.038
Neonatal aspiration syndrome						
Total	0.008 (0.089)	0.009 (0.094)	0.007 (0.086)	−0.001	(−0.003, 0.000)	.121
Male	0.008 (0.088)	0.008 (0.090)	0.007 (0.086)	−0.001	(−0.003, 0.001)	.492
Female	0.008 (0.091)	0.009 (0.097)	0.007 (0.087)	−0.002	(−0.004, 0.001)	.125

Notes: An ordinary least squares regression is used to compare the inpatient admission rates for infants born in 2015 versus 2017

^**^p < .05

^***^p < .01

In Additional file 1: Tables 1 and 2, we changed the outcome variable to a dummy that equals one if the individual had ever had an inpatient visit. The results are similar to the main analysis.

### Inpatient costs

Table 4 presents inpatient admission cost patterns. The average inpatient admission cost was 8412.3 RMB ($1320.61) (SD = 15,088.2), with 8484.1 RMB ($1331.88) (SD = 15,429.1) in 2015 and 8375.9 RMB ($1314.90) (SD = 14,913.0) in 2017. There was no change in inpatient costs per admission. However, among the five most commonly diagnosed diseases, there was an increase in inpatient costs for jaundice by 5.8% (*e*^0.056^-1) (coef = 0.056, CI = 0.016 to 0.096, *p* = .006).

**Table 4:** Total spending on inpatient admission

	Total	2015	2017	2017 versus 2015 (log spending)		
	Mean (SD)	Mean (SD)	Mean (SD)	Estimate	CI	*p*
Total	8412.3 (15,088.2)	8484.1 (15,429.1)	8375.9 (14,913.0)	0.023	(−0.010, 0.056)	.168
Whether infectious disease						
Infectious	7441.4 (9258.8)	7290.6 (8852.0)	7515.0 (9452.0)	0.052	(−0.003, 0.108)	.064
Noninfectious	8729.3 (16,540.9)	8861.1 (16,969.1)	8661.7 (16,317.6)	0.012	(−0.027, 0.052)	.542
Top 5 diseases						
Jaundice	4164.9 (4499.4)	4127.9 (5210.7)	4182.8 (4113.0)	0.056***	(0.016, 0.096)	.006
Pneumonia	7695.1 (9300.4)	7578.1 (8548.2)	7747.3 (9619.3)	0.042	(−0.028, 0.111)	.242
Preterm birth/small for gestational age	18,079.0 (23,667.5)	18,225.9 (24,583.4)	17,990.9 (23,111.2)	0.001	(−0.082, 0.084)	.983
Bronchitis	4923.5 (4160.2)	4989.8 (4855.3)	4872.5 (3545.1)	0.043	(−0.075, 0.161)	.472
Neonatal aspiration Syndrome	8109.5 (8309.6)	7942.1 (7501.0)	8224.0 (8834.2)	0.037	(−0.076, 0.149)	.519

Notes: An ordinary least squares regression is used to compare the spending associated with inpatient admission for infants born in 2015 versus 2017

^**^p < .05

^***^p < .01

Additional file 1: Fig. 1 presents out-of-pocket costs as a share of the total cost. Out-of-pocket costs represented 53.0% of all costs across all diseases, with jaundice having the highest share of out-of-pocket costs (57.4%) and preterm births/small for gestational age having the lowest share (51.0%).

The distribution of various medical costs by disease type is presented in Additional file 1: Fig. 2. The highest share was for checkups, accounting for 28.4% of total medical costs at RMB 8412.3 ($1320.61) (SD = 15,088.2), followed by treatment at 27.8%, other costs at 27.0%, medicine at 15.4%, and operations and procedures at 1.4%. Similar patterns are evident for different diseases.

### Other inpatient admission outcomes

Additional file 1: Figs. 3 and 4 presents descriptive data on other inpatient outcomes. The average length of hospital stay was 7.3 days, with hospitalizations for preterm births/infants small for their gestational age having the highest average length of stay (12.5 days) and hospitalizations for jaundice having the lowest average length of stay (5.0 days). The probability of going to a tertiary hospital was 76.2%, with infants hospitalized for neonatal aspiration syndrome having the highest rate (94.3%) and infants hospitalized for bronchitis having the lowest rate (56.5%).

## Discussion

### Principal findings

Based on URBMI data, this study provides an estimate of changes in inpatient admission rates and associated costs of infants between 0 and 6 months old after the universal two-child policy was implemented in China. The study yielded three main findings. First, inpatient admission rates were 24.9 and 29.8% for infants within 1 month and 6 months old, respectively. In comparison with 2015, inpatient rates increased among infants in both age groups, with larger increases observed among male infants. Second, compared with 2015, in 2017, there was an increase in inpatient admissions rates overall and for both infectious and noninfectious diseases. The leading five causes of hospitalization were jaundice, pneumonia, preterm birth or small size for gestational age, bronchitis, and neonatal aspiration syndrome. Inpatient admission rates were higher in 2017 than in 2015 for jaundice, pneumonia, lower for bronchitis, but not for preterm birth/small size for gestational age or neonatal aspiration syndrome. Third, the average cost per admission was RMB 8412.3 ($1320.61), with 53.0% percent paid out of pocket, and 76.2% of hospital stays took place in tertiary hospitals. There was no increase in spending per visit overall except for jaundice.

This study adds to our understanding of how the universal two-child policy affects infant inpatient outcomes in China. First, it provides an assessment of inpatient outcomes for infants up to 6 months of age. Second, it captures all types of infant hospitalizations and presents leading causes of hospitalization, providing a more completed understanding of infants’ healthcare utilization. Prior literature has examined only a limited number of infant health complications or diseases [5, 9]. Our results are consistent with the two major studies on this topic [5, 9]. More specifically, Li et al. [5] found an increase in maternal age but no increase in preterm births following the universal two-child policy, while Zhang et al. [9] found an increase in women with advanced maternal age and an increase in birth defects. The null result in preterm births is puzzling. Li et al. [5] did not provide a discussion on why there was no increase in preterm births despite the increase in advanced maternal age. It is possible that the universal two-child policy is associated with an increase in miscarriage or stillbirths or preterm that have resulted in neonatal death, and thus they do not show up in our sample. A dataset that covers information on miscarriage, stillbirths, and neonatal death is needed to examine this issue further. Examining infant hospitalization trends and understanding why infants are hospitalized before and after the universal two-child policy is critical to inform clinical practices and health policies governing the allocation of hospital resources. As there is an urgent shortage of pediatricians in China, to better meet the increasing healthcare needs of this cohort of children, an increased supply of pediatricians, continuous professional training and retention programs, and structural reforms to the pediatric care system are needed [9].

Consistent with previous studies [14, 15], we found that male infants were at a higher risk of being hospitalized and that the increase in inpatient admission rates was higher among males than among females. One explanation for these differences is biological: male infants are more vulnerable to illness, especially at a younger age [16]. Alternatively, possibly because of the old age security motive [17, 18], as well as patriarchal and son-preference culture in China [19], families are more likely to take male infants to hospitals [20]. Also, because mothers who gave birth following the universal two-child policy were older [5], and were more likely to give birth to male children as their second child [21], male children born after the two-child policy may be less healthy than male children born before the two-child policy. Thus, special attention should be paid to understanding and addressing gender differences in children’s health.

We find that the cost of an inpatient admission is not high in China. The average inpatient admission cost is RMB 8412.3 ($1320.61)—significantly lower than in the United States or other developed countries [22]. The average out-of-pocket cost is RMB 4458.5 ($699.92), which is not a catastrophic cost for most patients in China. The average annual household income in 2015 is RMB 31194.8 ($4897.14), and households’ total consumption expenditure net of food in 2015 is RMB 6359.7 ($998.38) in China [23]. The average hospitalization cost across all diseases is lower than the threshold for catastrophic health expenditures, defined as out-of-pocket payments amounting to greater than 40% of a household’s total consumption expenditures net of food [24, 25].

However, our findings are consistent with previous literature showing that the proportion of out-of-pocket costs for children is relatively high compared with that for adults [7]. Literature has shown that the share of costs paid out-of-pocket for all healthcare services is 28.8% in national data [26], whereas the share paid out-of-pocket for infants in our sample was 53.0%. Because the individuals in our sample are covered by URBMI, these differential out-of-pocket rates may be due to higher demand for services or drugs not included in the essential benefits package [7]. To reduce the burden posed by out-of-pocket healthcare costs, regulation and oversight of the efficient use of services should be implemented by the government. In addition, more research is needed to understand why hospitalization for infants results in a higher proportion of services or drugs that fall outside of the essential benefits package, as compared with adults. If these services or drugs are essential in treating infants, the depth and breadth of coverage need to be increased for infants to ensure financial protection for families.

There is evidence that spending is on the rise for jaundice after the universal two-child policy. Spending on the hospitalization cost of jaundice can increase due to an increase in the severity of diseases. Alternatively, as parents may have son preference due to old age security motive [17, 18], with a larger increase in jaundice for male infants (the associated coefficient is 0.027 in males versus 0.017 in females), spending on average may have increased because more male infants are hospitalized for jaundice. The increases in spending, as well as increases in the rate of inpatient care, add to economic burdens for families and for social health insurance. Thus, it is important to look for ways to contain costs.

In our analyses, we found that 76.2% of inpatient visits took place in tertiary hospitals. Even though China intends to build an integrated healthcare system, the system remains to be fragmented, and patients often bypass lower-tier hospitals, and tend to prefer tertiary hospitals because they are perceived to have higher quality [27]. This imbalance creates overwhelming demand at the tertiary level, with a single physician typically responsible for 80 to 100 visits per day in some tertiary children’s hospitals, while lower-level hospitals may be underutilized [28]. Because of the congestion, families with children often have to wait in long lines in seeking for care, and physicians can pay less attention to each case, causing tension between patients and physicians [29]. In addition, as dictated by the price schedule designed by the Ministry of Health, tertiary hospitals are more expensive than non-tertiary hospitals [30]. Thus, one important way to contain costs and reduce congestion in large hospitals is to strengthen the capacity of non-tertiary hospitals to treat infants.

### Policy implications

Understanding the hospitalization trends of infants is important for healthcare resource allocation and requires specific policies to aid the early diagnosis and treatment of preventable diseases. The increasing demand coupled with the acute shortage of pediatricians calls for an improvement in the quantity and quality of pediatric education and the workforce system. In addition, as male infants have a higher rate of inpatient admissions, special attention should be paid to understanding and addressing gender differences in children’s health.

While the rate of catastrophic cost is not high, the proportion of the out-of-pocket cost is higher than adults. In addition, there is an increase in spending on certain illnesses. Also, the rate of attending tertiary children’s hospitals is high. Thus, it is important to provide better financial protection for infants by increasing the depth and breadth of healthcare coverage while at the same time to strengthen the capacity of non-tertiary hospitals to treat infants to achieve the goal of containing costs and reducing congestion in large hospitals.

This paper also has important public policy implications in the era of the three-child policy. China announced in August 2021 that all couples are allowed to have up to three children. This policy has targeted parents who already have two children. If it is successful in encouraging families with two children to have a third, the likelihood of mothers being of advanced maternal age would be even higher as all these mothers have already given births to two children. With the well-established link between advanced maternal age and infant health problems [7, 8], it is possible that there would further increase in the hospitalization of infants post three-child policy. Thus, the healthcare system should monitor the health status of this cohort proactively, and adopt changes in response to the change in infant health.

### Limitations

Our findings should be cautiously interpreted due to several limitations. First, as there is no appropriate control group, we cannot rule out possible confounding factors and therefore cannot establish causality. There may be changes in the structure of the population of women of childbearing age even in absence of the universal two-child policy, thus biasing the result of our study. For example, the younger generation of women may be delaying the age of births regardless of population policy. Second, in our analyses, we compared healthcare utilization for infants born in 2015 versus those born in 2017. Because the universal two-child policy was announced in October 2015, births after June 2016 (9 months after the announcement of the universal two-child policy) are usually considered to fall in the range of the post-period. It is possible that the mothers who are successful in getting pregnant right after the announcement of the universal two-child period are younger, healthier, or have more resources, and our results may be different if we include this group of women. Because we do not have information on monthly births, we cannot answer this question empirically. Thus, the interpretation of the paper may be limited to the healthcare utilization of the cohort born in 2017. Second, our study is limited to the utilization of URBMI. If patients do not apply for health insurance or do not use health insurance, their hospitalizations do not appear in our dataset. However, because of the nontrivial cost associated with hospitalization and families’ ability to apply and use health insurance retroactively, the likelihood of hospitalization without health insurance for infants who are qualified for health insurance may be low. Third, inaccuracy and incompleteness of patients’ medical information may affect the accuracy of our analysis. Fourth, we do not have information on infant death, so our results are conditional on living infants. We also lack data on outpatient visits, so we cannot provide an estimate of total medical costs. Lastly, the study only uses data from one metropolitan city, and results from other regions of China are likely to differ.

## Conclusion

Changes in the utilization and cost of inpatient admissions among infants after the implementation of China’s universal two-child policy suggest that changes in fertility policies are associated with changes in infant health. An accurate understanding of healthcare utilization and expenditures in this new cohort is necessary to guide research priorities and investments in intervention strategies in an era of fertility control relaxation. Further studies on the long-term effects of birth policy changes on children’s need for healthcare utilization are also needed. This study also highlights the need for understanding the utilization of healthcare as China ushers in a universal three-child policy.

## Supplementary Information

**Additional file 1: Table 1.** Inpatient ever admission rates within 1 month and 6 months of birth. **Table 2.** Inpatient ever admission rates by disease (top 5 diseases). **Figure 1.** Out-of-pocket and reimbursed spending as a share of total spending for inpatient admission. **Figure 2.** Cost distribution by disease (top 5 diseases). **Figure 3.** Length of stay by disease (top 5 diseases). **Figure 4.** Tertiary hospital rate by disease (top 5 diseases).

## Data Availability

The datasets generated and/or analyzed during the current study are from China Health Insurance Research Association, which are not publicly available. The authors obtained the data through a request with permission from the China Health Insurance Research Association for research purposes. For questions or requests for the datasets, please contact the Center of Health Statistics and Information, National Health Commission of the People’s Republic of China, http://www.mohrss.gov.cn.

## Abbreviations

URBMI: Urban and Rural Basic Medical Insurance
UEBMI: Urban Employee Basic Medical Insurance
