# Supplementary material for: Estimates of inpatient admission rates and associated costs for infants before and after China’s universal two-child policy
Source: BMC Health Serv Res. 2022 Feb 8;22:163. doi: 10.1186/s12913-022-07571-9 (PMC8826662; doi:10.1186/s12913-022-07571-9)
Supplement: Supplementary file 1 — Additional file 1: Table 1. Inpatient ever admission rates within 1 month and 6 months of birth. Table 2. Inpatient ever admission rates by disease (top 5 diseases). Figure 1. Out-of-pocket and reimbursed spending as a share of total spending for inpatient admission. Figure 2. Cost distribution by disease (top 5 diseases). Figure 3. Length of stay by disease (top 5 diseases). Figure 4. Tertiary hospital rate by disease (top 5 diseases). [file 12913_2022_7571_MOESM1_ESM.docx]

**Additional file**

Table 1. Inpatient ever admission rates within 1 month and 6 months of birth

|  | Total | 2015 | | 2017 | | 2017 versus 2015 | | | | |
| --- | --- | --- | --- | --- | --- | --- | --- | --- | --- | --- |
|  | Mean (SD) | Mean (SD) | | Mean (SD) | | Estimate | | CI | | *p* |
| Total |  |  | |  | |  | |  | |  |
| Within 1 month | 0.217 | 0.196 | | 0.230 | | 0.034*** | | (0.026, 0.041) | | <.001 |
|  | (0.412) | (0.397) | | (0.421) | |  | |  | |  |
| Within 6 months | 0.244 | 0.222 | | 0.257 | | 0.035*** | | (0.027, 0.043) | | <.001 |
|  | (0.430) | (0.416) | | (0.437) | |  | |  | |  |
| *N* | 246,040 | 90,595 | | 155,445 | |  | |  | |  |
| Male |  |  | |  | |  | |  | |  |
| Within 1 month | 0.228 | 0.201 | | 0.244 | | 0.044*** | | (0.033, 0.054) | | <.001 |
|  | (0.420) | (0.401) | | (0.430) | |  | |  | |  |
| Within 6 months | 0.260 | 0.232 | | 0.277 | | 0.046*** | | (0.035, 0.057) | | <.001 |
|  | (0.439) | (0.422) | | (0.448) | |  | |  | |  |
| *N* | 131,275 | 49,325 | | 81,950 | |  | |  | |  |
| Female |  |  | |  | |  | |  | |  |
| Within 1 month | 0.205 | 0.191 | | 0.213 | | 0.023*** | | (0.012, 0.034) | | <.001 |
|  | (0.404) | (0.393) | | (0.410) | |  | |  | |  |
| Within 6 months | 0.226 | 0.211 | | 0.234 | | 0.023*** | | (0.012, 0.035) | | <.001 |
|  | (0.418) | (0.408) | | (0.424) | |  | |  | |  |
| *N* | 114,765 | 41,270 | | 73,495 | |  | |  | |  |
| Notes: An ordinary least squares regression is used to compare the inpatient admission rates for infants born in 2015 versus 2017. | | | | | | | | | | |
| ***p* < .05. ****p* < .01. | | |  | |  | |  | |  | |

Table 2. Inpatient ever admission rates by disease (top 5 diseases)

|  | Total | 2015 | 2017 | 2017 versus 2015 | | |
| --- | --- | --- | --- | --- | --- | --- |
|  | Mean (SD) | Mean (SD) | Mean (SD) | Estimate | CI | *p* |
| Jaundice |  |  |  |  |  |  |
| Total | 0.109 | 0.096 | 0.116 | 0.020*** | (0.014, 0.025) | <.001 |
|  | (0.311) | (0.295) | (0.320) |  |  |  |
| Male | 0.113 | 0.097 | 0.123 | 0.026*** | (0.018, 0.034) | <.001 |
|  | (0.317) | (0.296) | (0.328) |  |  |  |
| Female | 0.104 | 0.096 | 0.108 | 0.013*** | (0.004, 0.021) | .003 |
|  | (0.305) | (0.294) | (0.311) |  |  |  |
| Pneumonia |  |  |  |  |  |  |
| Total | 0.042 | 0.035 | 0.045 | 0.010*** | (0.006, 0.013) | <.001 |
|  | (0.200) | (0.185) | (0.208) |  |  |  |
| Male | 0.048 | 0.041 | 0.053 | 0.012*** | (0.006, 0.017) | <.001 |
|  | (0.215) | (0.199) | (0.224) |  |  |  |
| Female | 0.034 | 0.028 | 0.037 | 0.008*** | (0.003, 0.013) | <.001 |
|  | (0.180) | (0.166) | (0.188) |  |  |  |
| Preterm birth/small for gestational age | | |  |  |  |  |
| Total | 0.037 | 0.037 | 0.036 | -0.000 | (-0.004, 0.003) | .809 |
|  | (0.188) | (0.188) | (0.187) |  |  |  |
| Male | 0.037 | 0.036 | 0.037 | 0.001 | (-0.003, 0.006) | .566 |
|  | (0.188) | (0.186) | (0.189) |  |  |  |
| Female | 0.037 | 0.038 | 0.036 | -0.003 | (-0.008, 0.003) | .330 |
|  | (0.188) | (0.192) | (0.185) |  |  |  |
| Bronchitis |  |  |  |  |  |  |
| Total | 0.008 | 0.009 | 0.007 | -0.002** | (-0.003, -0.000) | .034 |
|  | (0.087) | (0.093) | (0.084) |  |  |  |
| Male | 0.009 | 0.010 | 0.009 | -0.001 | (-0.004, 0.001) | .245 |
|  | (0.097) | (0.101) | (0.094) |  |  |  |
| Female | 0.006 | 0.007 | 0.005 | -0.002 | (-0.004, 0.000) | .060 |
|  | (0.075) | (0.083) | (0.070) |  |  |  |
| Neonatal aspiration syndrome | | |  |  |  |  |
| Total | 0.008 | 0.009 | 0.007 | -0.001 | (-0.003, 0.000) | .125 |
|  | (0.088) | (0.093) | (0.086) |  |  |  |
| Male | 0.008 | 0.008 | 0.007 | -0.001 | (-0.003, 0.001) | .492 |
|  | (0.088) | (0.090) | (0.086) |  |  |  |
| Female | 0.008 | 0.009 | 0.007 | -0.002 | (-0.004, 0.001) | .129 |
|  | (0.089) | (0.096) | (0.085) |  |  |  |
| Notes: An ordinary least squares regression is used to compare the inpatient admission rates for infants born in 2015 versus 2017. | | | | | | |
| ***p* < .05. ****p* < .01. | | |  |  |  |  |

Figure 1. Out-of-pocket and reimbursed spending as a share of total spending for inpatient admission

Figure 2. Cost distribution by disease (top 5 diseases)

Figure 3. Length of stay by disease (top 5 diseases)

Figure 4. Tertiary hospital rate by disease (top 5 diseases)
